# Systemic immune-inflammation index may predict the acute kidney injury and prognosis in patients with spontaneous cerebral hemorrhage undergoing craniotomy: a single-center retrospective study

**DOI:** 10.1186/s12882-023-03124-2

**Published:** 2023-03-25

**Authors:** Qiang Wang, Shifang Li, Meifeng Sun, Junwei Ma, Jian Sun, Mingchao Fan

**Affiliations:** 1 Department of Nephrology, Qilu Hospital (Qingdao), Cheeloo College of Medicine, Shandong University, Qingdao, China; 2 Department of Neurosurgery, the Affiliated Hospital of Qingdao University, Qingdao, 266003 China; 3 Department of Traditional Chinese Medicine, the Affiliated Hospital of Qingdao University, Qingdao, China; 4 Department of Neurosurgical Intensive Care Unit, the Affiliated Hospital of Qingdao University, Qingdao, China

**Keywords:** Acute kidney injury, Spontaneous cerebral hemorrhage, Craniotomy, Systemic immune-inflammation index, Systemic inflammation response index

## Abstract

**Background:**

The systemic immune-inflammation index (SII) is an emerging prognostic marker of cancer. We aimed to explore the predictive ability of the SII on acute kidney injury (AKI) and prognosis in patients with spontaneous cerebral hemorrhage (SCH) who underwent craniotomy.

**Methods:**

Patients with SCH who underwent craniotomy between 2014 and 2021 were enrolled in this study. The epidemiology and predictive factors for AKI after SCH were analyzed. The prognostic factors for clinical outcomes in patients with SCH and AKI were further investigated. The prognostic factors were then analyzed using a logistic regression model and a receiver operating characteristic curve.

**Results:**

In total, 305 patients were enrolled in this study. Of these, 129 (42.3%) patients presented with AKI, and 176 (57.7%) patients were unremarkable. The SII (odds ratio [OR], 1.261; 95% confidence interval [CI], 1.036–1.553; *P* = 0.020) values and serum uric acid levels (OR, 1.004; 95% CI, 1.001–1.007; *P* = 0.005) were significant predictors of AKI after SCH craniotomy. The SII cutoff value was 1794.43 (area under the curve [AUC], 0.669; 95% CI, 0.608–0.730; *P* < 0.001; sensitivity, 65.9%; specificity, 65.1%). Of the patients with AKI, 95 and 34 achieved poor and good outcomes, respectively. SII values (OR, 2.667; 95% CI, 1.167–6.095; *P* = 0.020), systemic inflammation response index values (OR, 1.529; 95% CI, 1.064–2.198; *P* = 0.022), and Glasgow Coma Scale (GCS) scores on admission (OR, 0.593; 95% CI, 0.437–0.805; *P* = 0.001) were significant in the multivariate logistic regression analysis. The cutoff SII value was 2053.51 (AUC, 0.886; 95% CI, 0.827–0.946; *P* < 0.001; sensitivity, 78.9%; specificity, 88.2%).

**Conclusions:**

The SII may predict AKI in patients with SCH who underwent craniotomy and may also predict the short-term prognosis of these patients.

## Background

Spontaneous cerebral hemorrhage (SCH) is a devastating disease that accounts for 10–15% of stroke cases and is associated with high morbidity and mortality rates [1]. Craniotomy is the most common method to evacuate hematoma, resolve brain pressure, and improve neurological function. Simultaneously, craniotomy is required in the following conditions: larger hematoma, higher intracranial pressure, more severe conditions, and presence or increased risk of cerebral hernia [2]. The high incidence of SCH is attributed to the large number of patients with hypertension and diabetes mellitus. These underlying diseases indicate a history of vascular injury. The administration of dehydrating agents, antibiotics, and contrast media, hemodynamic variations, and post-surgery pain management add more challenges to the kidney, rendering them more prone to acute kidney injury (AKI). AKI is a common complication, occurring in as high as 20% of SCH cases, and AKI superimposed on SCH is associated with even worse clinical outcomes [3, 4]. Therefore, exploration of markers to predict or indicate AKI after SCH is warranted.

The systemic immune-inflammation index (SII), calculated using lymphocytes, neutrophils, and platelets, is an emerging marker that has been emphasized in several clinical scenarios. The SII was initially developed to reflect the balance of host inflammatory and immune status, which could predict the outcome in patients with hepatocellular carcinoma [5]. Recently, it has been investigated as a prognostic factor for various cancers [6, 7], inflammatory status [8, 9], and coronary disease [10]. The systemic inflammation response index (SIRI), which is also calculated from peripheral blood cells, has similar characteristics to those of the SII [8, 9].

AKI is also a local inflammatory response characterized by the infiltration of neutrophils, monocytes, and lymphocytes in the renal parenchyma [11, 12]. The infiltrated cells originate from peripheral hemocytes [13–15]. Patients who undergo surgery have a high neutrophil-to-lymphocyte ratio due to increased secretion of stress hormones, resulting in neutrophil margination in the bloodstream with redistribution of lymphocytes into lymphatic tissues [16]. The SII is a marker for AKI in other clinical settings [17, 18]. To date, the SII has not been reported as a predictive factor for AKI in patients with SCH who undergo craniotomy. Although the mortality rate is striking, the specific prognostic factors among patients with post-craniotomy AKI are unknown. In the present study, we aimed to determine whether the SII can predict AKI in patients with SCH after craniotomy and to investigate whether it can be a prognostic marker in patients with AKI.

## Methods

### Study population and data collection

**Fig. 1:**
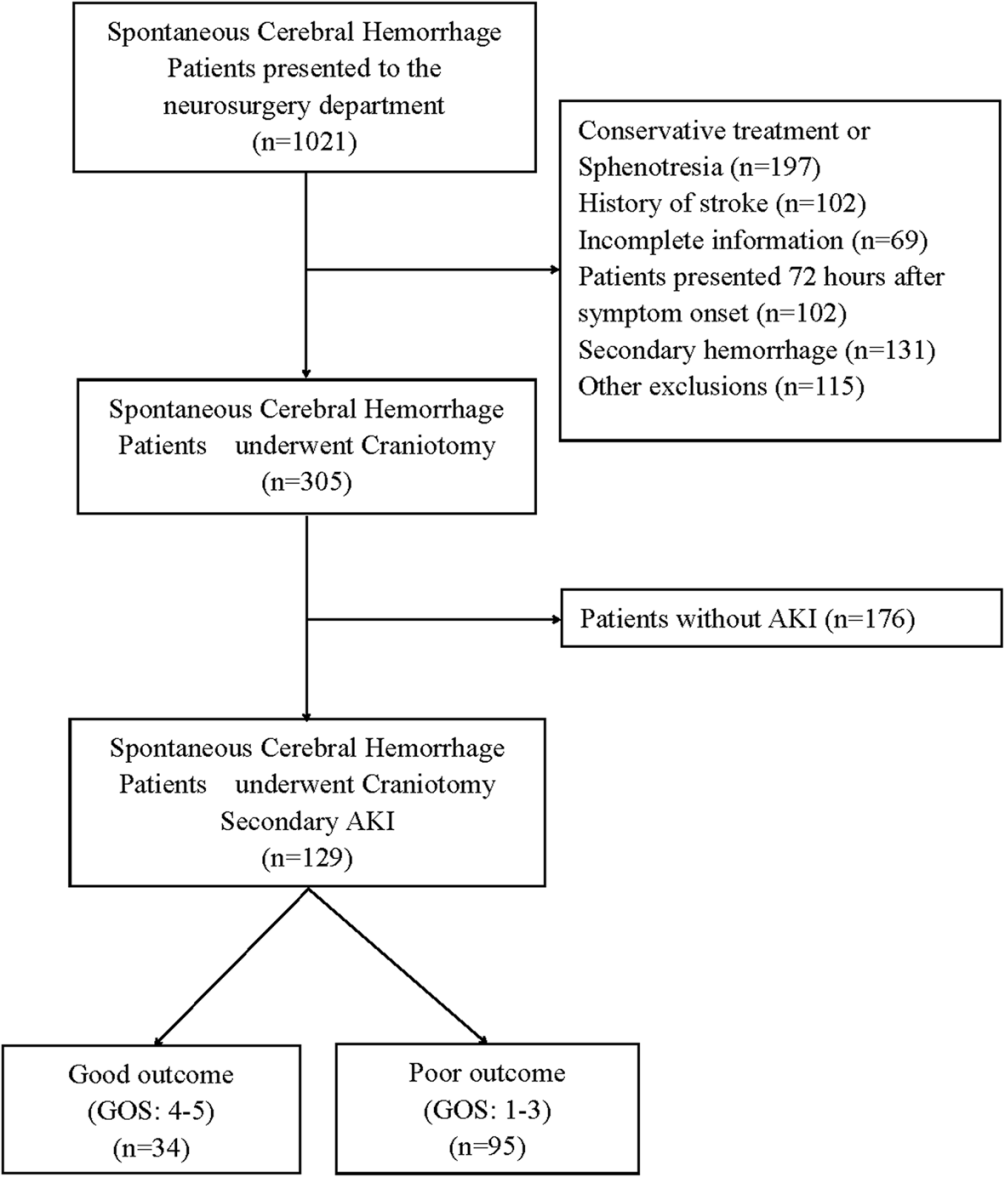
The study flowchart. AKI, acute kidney injury; GOS, Glasgow Outcome Scale

We retrospectively collected data from patients who underwent craniotomy for SCH and were diagnosed with AKI between October 2014 and June 2021 at the Affiliated Hospital of Qingdao University, Qingdao, China. Clinical variables were extracted from the scientific research big data platform and hospital information system. Preoperative laboratory data within 72 h were analyzed and calculated. The exclusion criteria were as follows: (1) secondary SCH (trauma, aneurysm, vascular malformation, hemorrhagic transformation, or tumor); (2) acute and/or chronic inflammatory status, hematological or autoimmune diseases, or cancers; (3) proteinuria, glomerulonephritis, chronic kidney disease (CKD), or obstructive nephropathy; and (4) incomplete information (Fig. 1). Craniotomy was performed within 72 h of the initial symptoms. All patients received consistent management. Mannitol was administered intravenously at a dose of 0.5 g/kg body weight every 6 h until cerebral edema was relieved. The enrolled patients were dichotomized into the AKI and non-AKI groups. Based on the outcomes, patients with AKI were further grouped as having poor or good outcomes. This study was approved by the Institutional Review Board (IRB) of the Affiliated Hospital of Qingdao University (IRB reference no: QYFY-WZLL-26808). All methods were performed in accordance with the Declarations of Helsinki.

### Study definitions

SII values were calculated as follows: platelet count × neutrophil count/lymphocyte count. SIRI values were calculated as follows: neutrophil count × monocyte count/lymphocyte count. The SII and SIRI values were calculated using preoperative data. The baseline serum creatinine (SCr) level was evaluated on admission and re-evaluated at 12, 24, and 48 h after the operation. AKI was defined according to the 2012 Kidney Disease: Improving Global Outcomes classification [19] as any of the following: increase in SCr level by ≥0.3 mg/dl (26.5 μmol/l) within 48 h or increase in SCr level to ≥1.5 times baseline within the prior 7 days or urine volume < 0.5 ml/kg/h for 6 h. AKI was staged according to the following criteria: (1) stage 1, increase in SCr level to 1.5–1.9 times baseline or ≥ 0.3 mg/dl (26.5 μmol/l) or urine output < 0.5 ml/kg/h for 6–12 h; stage 2, increase in SCr to 2.0–2.9 times baseline or < 0.5 ml/kg/h for ≥12 h; and stage 3, increase in SCr level to 3.0 times baseline or to ≥4.0 mg/dl (353.6 μmol/l) or initiation of renal replacement therapy or urine output < 0.3 ml/kg/h for ≥24 h or anuria for ≥12 h. Proteinuria was defined as > 1+ in the routine urine test or a total protein-to-creatinine ratio > 0.15. Glomerulonephritis was defined as proteinuria, hematuria, and/or hypertension with or without renal pathological evidence. CKD was defined as abnormalities in kidney structure or function for > 3 months. Obstructive nephropathy was defined as anatomical and functional abnormalities of the urinary tract based on imaging evidence.

The operation indications were as follows: (1) estimated hematoma volume > 30 ml, (2) evident mass effect, and (3) mass effect causing a shift of midline structures [20, 21]. Hematoma volume for surgical indication was assessed by brain computed tomography using the formula ABC/2 [22]. Glasgow Coma Scale (GCS) scores were calculated by evaluating the eye-opening, verbal, and motor responses [23]. The Glasgow Outcome Scale (GOS) score was determined using five categories: good recovery (score, 5), moderate disability (score, 4), severe disability (score, 3), vegetative state (score, 2), and dead (score, 1) [24]. The outcomes of 30-day post craniotomy were evaluated using GOS. A total score of 1–3 was defined as a poor outcome, whereas 4–5 indicated a good outcome.

### Statistical analyses

Statistical analyses were performed using the IBM SPSS Statistics version 24.0. Continuous variables are summarized as mean ± standard deviation. The abnormal distribution of continuous variables is summarized as median and interquartile ranges (25th–75th percentile). Categorical data are presented as frequencies and percentages. Student’s t-test and Kruskal–Wallis test were used to compare the normal and abnormal distributions of continuous variables, respectively. The chi-squared test was used to compare categorical data. Logistic regression analysis was performed to identify the independent risk factors. Receiver operating characteristic (ROC) curve analysis was performed to determine the sensitivity, specificity, and differences in the area under the curve (AUC). A *P* value < 0.05 was considered statistically significant.

## Results

### Demographic and baseline characteristics

The basic information of all the patients is presented in Table 1. In total, 305 patients were enrolled in this study. Of these, 129 (42.3%) patients with AKI were allocated to the AKI group, and 176 (57.7%) patients were allocated to the non-AKI group (Fig. 1). Among these patients with AKI, stage 1, 2, and 3, AKIs were observed in 103 (79.84%), 22 (17.05%), and 4 (3.1%) patients, respectively. Only one patient received renal replacement therapy. Of these 305 patients, 24 died, 15 were in the AKI group, and 9 were in the non-AKI group (χ^2^ = 4.357, *P* = 0.037).

**Table 1:** The demographic and baseline characteristics of patients in the AKI and non-AKI groups

Variable	Total (*n* = 305)	AKI		*P*-value
		YES (*n* = 129)	NO (*n* = 176)	
Sex (female)	91 (29.84)	34 (26.36)	57 (32.39)	0.256
Age (years)	56 (47–65)	56 (46–66)	57 (47–65)	0.972
BMI	25.35 (23.30–27.55)	25.50 (23.30–27.75)	24.95 (23.19–27.50)	0.388
Smoking (%)	79 (25.90)	36 (27.91)	43 (24.43)	0.494
Drinking (%)	86 (28.20)	35 (27.13)	51 (28.98)	0.723
Hypertension (%)	195 (63.93)	82 (63.57)	113 (64.20)	0.909
Diabetes (%)	24 (7.87)	11 (8.53)	13 (7.39)	0.715
Systolic Pressure (mmHg)	157 (140–180)	160 (141–190)	154 (137–178)	**0.038**
Diastolic Pressure (mmHg)	91 (80–105)	91 (80–107)	92 (80–100)	0.801
Heart Rate (Times/Min)	80 (73–90)	81 (74–93)	80 (72–89)	0.057
Pre-admission Aspirin (%)	41 (13.44)	22 (17.05)	19 (10.80)	0.113
Pre-admission ACEI (%)	29 (9.51)	14 (10.85)	15 (8.52)	0.493
Postoperative Cephalosporins (%)	167 (54.75)	76 (58.91)	91 (51.70)	0.211
Postoperative Vancomycin (%)	36 (11.80)	19 (14.73)	17 (9.66)	0.175
Postoperative Penicillins (%)	102 (33.44)	40 (31.01)	62 (35.23)	0.440
Postoperative Furosemide* (%)	135 (44.26)	63 (48.84)	72 (40.91)	0.168
Length of ICU stay (days)	3 (1–9)	4 (1–13)	2 (0–6)	**0.002**
Length of stay (days)	17 (12–26)	17 (12–27)	16 (11–25)	0.312
GCS on Admission	9 (7–12)	9 (7–12)	9 (8–12)	0.068
Creatinine on Admission (umol/L)	62.97 (52.60–76.00)	61.00 (50.80–79.00)	64.81 (54.20–75.73)	0.227
Creatinine at diagnosis of AKI (umol/L)	85.70 (70.76–101.31)	97.00 (78.50–121.45)	80.00 (66.56–91.00)	**< 0.001**
eGFR (ml/min)	104.15 (90.42–115.31)	104.32 (89.28–113.78)	104.12 (91.53–117.82)	**0.354**
Hematoma Volume (mL)	50 (40–80)	50 (50–80)	50 (40–70)	**0.021**
Intraventricular Hematoma (%)	135 (44.26)	64 (49.61)	71 (40.34)	0.107
underwent angiogram (%)	8 (2.62)	3 (2.33)	5 (2.84)	1.000
Serum K^+^ (mmol/L)	3.8 (3.4–4.1)	3.7 (3.4–4.1)	3.8 (3.5–4.1)	0.166
Serum Na^+^ (mmol/L)	140 (137–142)	140 (137–142)	140 (137–142)	0.562
Serum Cl^−^ (mmol/L)	102 (100–105)	103 (99–105)	102 (100–105)	0.760
Monocyte (×10^9^/L)	0.54 (0.37–0.75)	0.52 (0.35–0.74)	0.56 (0.38–0.77)	0.701
Neutrophil (× 10^9^/L)	9.67 (6.63–12.86)	11.29 (7.86–14.62)	8.55 (5.80–11.27)	**< 0.001**
Lymphocyte (×10^9^/L)	1.16 (0.77–1.78)	0.98 (0.67–1.45)	1.28 (0.87–1.96)	**< 0.001**
Leukocyte (×10^9^/L)	11.68 (8.99–14.86)	12.85 (9.97–16.38)	10.82 (8.78–13.36)	**< 0.001**
Hemoglobin (g/L)	144.39 ± 17.97	146.93 ± 19.21	142.53 ± 16.82	**0.038**
Platelet (×10^9^/L)	211 (176–253)	212 (178–258)	208 (175–250)	0.745
SIRI	4.05 (2.04- = 7.69)	5.43 (2.74–11.25)	3.60 (1.45–6.21)	**< 0.001**
SII	1730.00 (988.26–3089.28)	2292.08 (1229.63–3586.18)	1478.36 (753.36–2570.82)	**< 0.001**
Blood Glucose (mmol/L)	7.80 (6.80–9.16)	8.19 (6.98–9.87)	7.60 (6.43–8.72)	**0.008**
Serum Uric Acid (μmol/L)	225 (167–286)	249 (188–307)	207 (152–260)	**0.001**
D-Dimer (μg/L)	390 (270–640)	370 (250–640)	400 (275–647)	0.510
Postoperative Bleeding and Reoperation (%)	14 (4.59)	7 (5.43)	7 (3.98)	0.550

*AKI* acute kidney injury, *BMI* body mass index, *GCS* Glasgow coma scale, *SIRI* systemic inflammation response index, *SII* systemic immune-inflammation index

### Association between systemic immune-inflammation index (SII) values and acute kidney injury (AKI)

**Fig. 2:**
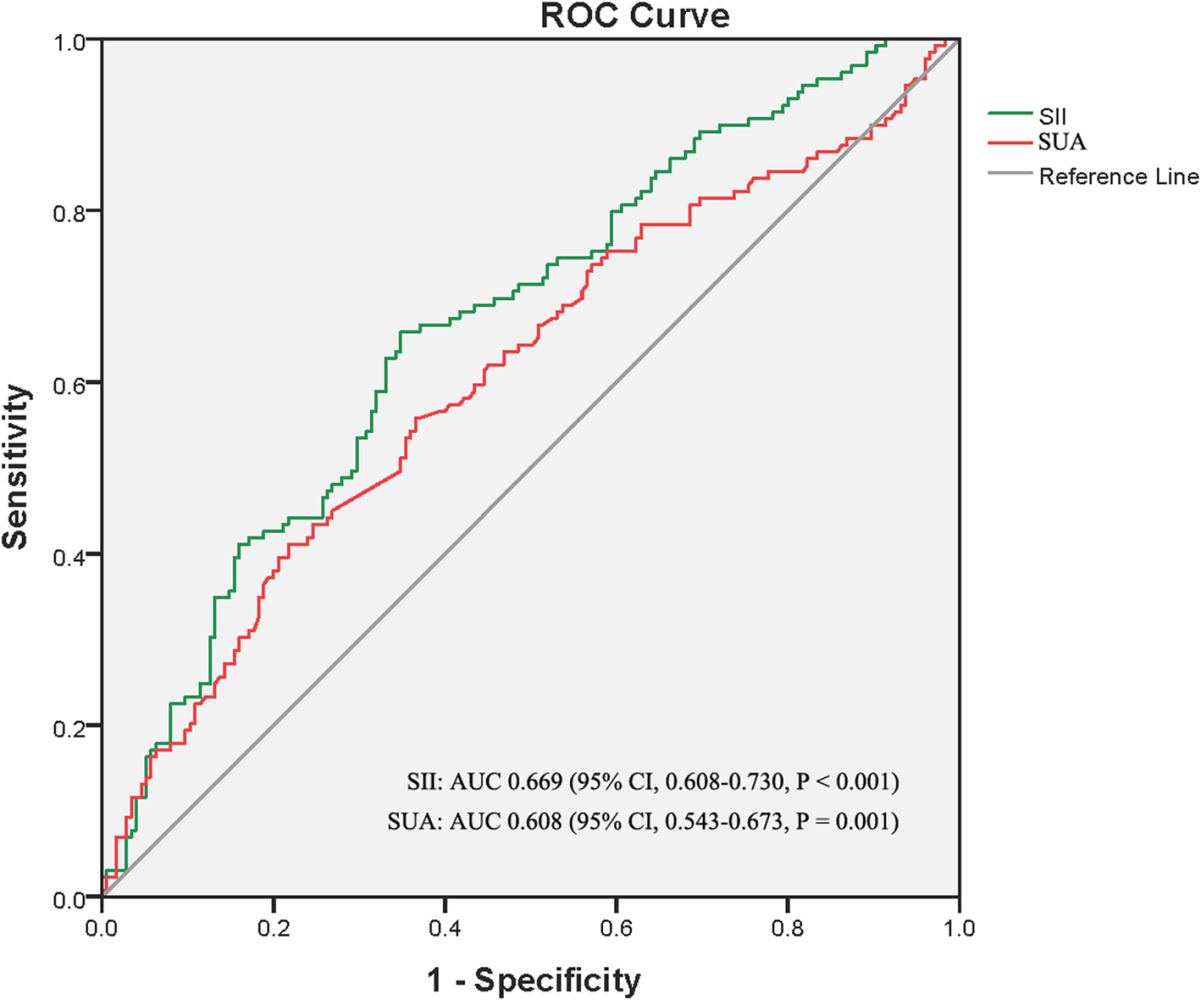
The receiver operating characteristic curves and area under the curves of the systemic immune inflammation index values and serum uric acid levels for predicting acute kidney injury in patients with spontaneous cerebral hemorrhage post craniotomy

The baseline characteristics of the AKI and non-AKI groups were compared using univariate analysis. We found that systolic pressure; hematoma volume; neutrophil, lymphocyte, and leukocyte counts; hemoglobin level; SII and SIRI values; and blood glucose and serum uric acid (SUA) concentrations were statistically different between the two groups (Table 1). These variables, except the length of intensive care unit (ICU) stay and neutrophil, lymphocyte, and leukocyte counts, were analyzed using univariate and binary logistic regressions (Table 2). They were statistically significant, except for systolic pressure in the univariate analysis, whereas only SII values (odds ratio [OR], 1.261; 95% confidence interval [CI], 1.036–1.553; *P* = 0.020) and SUA concentrations (OR, 1.004; 95% CI, 1.001–1.007; *P* = 0.005) were significant in the binary logistic regression. The discrimination ability of the SII and SUA was determined using an ROC curve. The AUCs for SII and SUA were 0.669 (95% CI, 0.608–0.730; *P* < 0.001) and 0.608 (95% CI, 0.543–0.673; *P* = 0.001), respectively (Fig. 2). The optimal cutoff value of the SII was 1794.43, with sensitivity and specificity of 65.9 and 41.1%, respectively. The optimal cutoff value of the SUA was 268.9, with sensitivity and specificity of 65.1 and 78.3%, respectively. To investigate which factor would predict the severity of AKI, the SII and SUA values were then inputted into a multivariate ordered logistic regression model. Neither SII nor SUA value could predict the severity of AKI.

**Table 2:** Univariate and multivariate regression analysis of factors related to AKI

Predictors	Univariate analysis		Multivariate analysis	
	OR (95% CI)	*P*-value	OR (95% CI)	*P*-value
Systolic Pressure (mmHg)	1.007 (1.000–1.015)	0.066		
Hematoma Volume (mL)	1.013 (1.003–1.023)	0.010	1.009 (0.998–1.020)	0.115
Hemoglobin (g/L)	1.014 (1.001–1.027)	0.036	1.010 (0.996–1.025)	0.166
SIRI	1.092 (1.046–1.140)	< 0.001	1.036 (0.979–1.097)	0.223
SII	1.365 (1.184–1.574)	< 0.001	1.261 (1.036–1.553)	**0.020**
Blood Glucose (mmol/L)	1.103 (1.016–1.196)	0.019	1.050 (0.962–1.146)	0.276
Serum Uric Acid (μmol/L)	1.004 (1.002–1.007)	0.001	1.004 (1.001–1.007)	**0.005**

*SIRI* systemic inflammation response index, *SII* systemic immune-inflammation index

### The predictive ability of the SII in the outcomes of AKI

Based on the GOS scores at 30-day post operation, the patients with AKI were dichotomized into two groups: poor (*n* = 95, 73.6%) or good (*n* = 34, 26.4%). The basic characteristics of the patients were compared using univariate analysis (Table 3). We found that the length of ICU stay; GCS score on admission; hematoma volume; monocyte, neutrophil, lymphocyte, and leukocyte counts; SIRI and SII values; and blood glucose and D-dimer levels were statistically different between the two groups. These variables, except length of ICU stay and monocyte, neutrophil, lymphocyte, and leukocyte counts, were then analyzed using univariate logistic regression. Significant variables were entered into a multivariate logistic regression model. GCS score on admission, hematoma volume, SII and SIRI values, and blood glucose level were statistically significant in univariate logistic regression, whereas only GCS score on admission (OR, 0.593; 95% CI, 0.437–0.805; *P* = 0.001), SII value (OR, 2.667; 95% CI, 1.167–6.095; *P* = 0.020), and SIRI value (OR, 1.529; 95% CI, 1.064–2.198; *P* = 0.022) were significant in the multivariate logistic regression analysis (Table 4).

**Table 3:** The demographic and baseline characteristics of patients in AKI patients

Variable	Total (*n* = 129)	AKI		*P*-value
		Unfavorable (*n* = 95)	Favorable (*n* = 34)	
Sex (female, %)	34 (26.36)	27 (28.42)	7 (20.59)	0.374
Age (years)	56 (46–66)	56 (47–67)	53 (46–66)	0.187
BMI	25.50 (23.31–27.75)	25.50 (23.40–27.68)	25.60 (22.80–28.48)	0.835
Smoking (%)	36 (27.91)	27 (28.42)	9 (26.47)	0.828
Drinking (%)	35 (27.13)	28 (29.47)	7 (20.59)	0.317
Hypertension (%)	82 (63.57)	64 (67.37)	18 (52.94)	0.134
Diabetes (%)	11 (8.53)	9 (9.47)	2 (5.88)	0.775
Systolic Pressure (mmHg)	164.40 ± 30.63	166.18 ± 32.23	159.41 ± 25.37	0.220
Diastolic Pressure (mmHg)	91 (80–107)	91 (80–110)	91 (82–107)	0.742
Heart Rate (Times/Min)	81 (75–93)	84 (76–96)	80 (70–89)	0.266
Length of ICU stay (days)	4 (1–13)	6 (2–15)	1 (0–3)	**< 0.001**
AKI stage				
Grade I (%)	103 (79.84)	69 (72.63)	34 (100.00)	**< 0.001**
Grade II (%)	22 (17.05)	22 (21.05)	0 (0.00)	**0.001**
Grade III (%)	4 (3.10)	4 (1.05)	0 (0.00)_	0.573
Creatinine on Admission (umol/L)	61.00 (50.80–79.00)	62.63 (51.10–86.60)	59.05 (48.51–72.11)	0.210
Creatinine at diagnosis of AKI (umol/L)	97.00 (78.50–121.45)	101.05 (86.70–135.15)	85.35 (71.25–112.25)	**0.026**
Length of stay (days)	17 (12–27)	18 (12–28)	15 (12–22)	0.169
GCS on Admission	9 (7–12)	8 (5–10)	12 (9–13)	**< 0.001**
Hematoma Volume (mL)	50 (50–80)	60 (50–80)	50 (44–60)	**0.005**
Intraventricular Hematoma (%)	64 (49.61)	51 (53.68)	13 (38.24)	0.122
Monocyte (×10^9^/L)	0.52 (0.35–0.74)	0.58 (0.38–0.85)	0.45 (0.27–0.61)	**0.007**
Neutrophil (×10^9^/L)	11.48 ± 4.59	12.88 ± 4.27	7.57 ± 2.91	**< 0.001**
Lymphocyte (× 10^9^/L)	0.98 (0.67–1.45)	0.84 (0.59–1.26)	1.31 (0.98–1.94)	**< 0.001**
Leukocyte (×10^9^/L)	13.33 ± 4.90	14.66 ± 4.74	9.61 ± 3.12	**< 0.001**
Hemoglobin (g/L)	146.93 ± 19.21	146.20 ± 20.83	148.97 ± 13.75	0.386
Platelet (×10^9^/L)	216.86 ± 64.17	217.01 ± 62.34	216.44 ± 70.02	0.967
SIRI	5.34 (2.82–11.44)	8.18 (4.72–12.36)	2.15 (1.26–3.22)	**< 0.001**
SII	2382.63 (1381.46–3627.25)	3150.09 (2142.30–4258.31)	1151.49 (726.28–1802.99)	**< 0.001**
Blood Glucose (mmol/L)	8.19 (6.99–9.88)	8.40 (7.23–10.34)	7.31 (6.38–9.10)	**0.002**
Serum Uric Acid (μmol/L)	249 (188–307)	246 (184–307)	250 (199–300)	0.696
D-Dimer (μg/L)	370 (250–640)	420 (280–680)	335 (180–585)	**0.047**
Postoperative Bleeding and Reoperation (%)	7 (5.43)	6 (6.32)	1 (2.94)	0.761

*AKI* acute kidney injury, *BMI* body mass index, *GCS* Glasgow coma scale, *SIRI* systemic inflammation response index, *SII* systemic immune-inflammation index

**Table 4:** Univariate and multivariate regression analysis of factors related to outcomes of AKI patients

Predictors	Univariate analysis		Multivariate analysis	
	OR (95% CI)	*P*-value	OR (95% CI)	*P*-value
GCS on Admission	0.637 (0.534–0.761)	< 0.001	0.593 (0.437–0.805)	**0.001**
Hematoma Volume (mL)	1.031 (1.009–1.053)	0.005	1.019 (0.987–1.052)	0.250
SIRI	1.935 (1.454–2.575)	< 0.001	1.529 (1.064–2.198)	**0.022**
SII	5.149 (2.700–9.818)	< 0.001	2.667 (1.167–6.095)	**0.020**
Blood Glucose (mmol/L)	1.367 (1.086–1.721)	0.008	1.018 (0.821–1.261)	0.872
D-Dimer (ng/mL)	1.001 (1.000–1.002)	0.085		

*SIRI* systemic inflammation response index, *SII* systemic immune-inflammation index

**Fig. 3:**
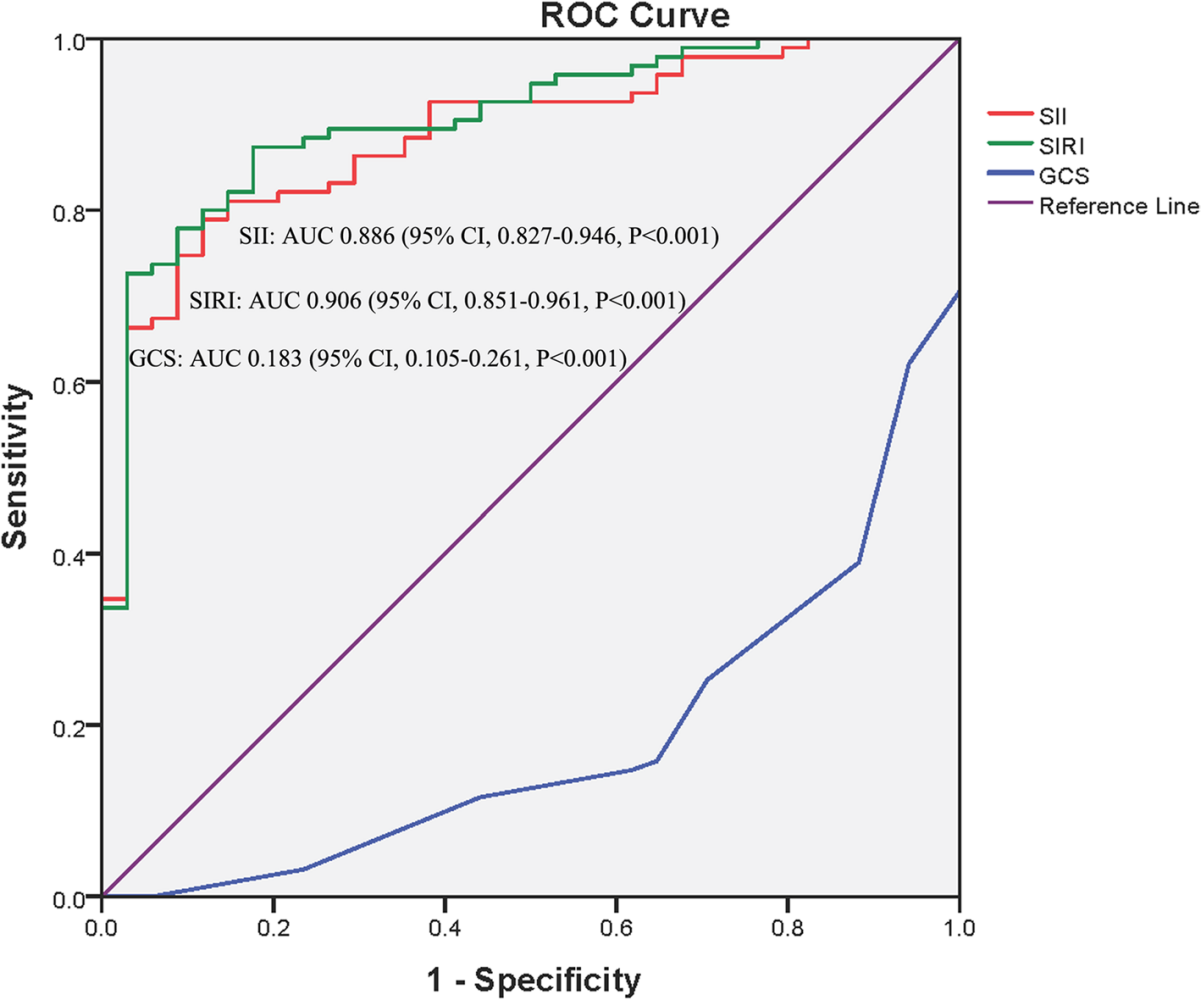
The receiver operating characteristic curves and the area under the curves of the systemic immune inflammation index and systemic inflammation response index levels and initial Glasgow Coma Scale scores for predicting poor outcomes in patients with acute kidney injury and spontaneous cerebral hemorrhage post craniotomy

ROC curves were constructed to evaluate the predictive ability of SII and SIRI values and GCS scores. The corresponding AUCs of the SII, SIRI, and GCS were 0.886 (95% CI, 0.827–0.946; *P* < 0.001), 0.906 (95% CI, 0.851–0.961; *P* < 0.001), and 0.183 (95% CI, 0.105–0.261; *P* < 0.001), respectively (Fig. 3). The optimal cutoff values for the SII, SIRI, and GCS were 2053.51, 3.48, and 8.5, respectively. The sensitivities and specificities were 78.9 and 88.2%, 87.4 and 82.4%, and 38.9 and 11.8%, respectively.

## Discussion

In the present retrospective study, we found that SII values on admission weakly predicted the development of AKI in patients with SCH who underwent craniotomy and that SII could predict the outcomes of these patients with AKI. A high incidence rate of AKI (42.3%) was observed in our cohort, which was consistent with other reports of neurological intensive scenarios [25, 26] and postoperative patients in ICUs [27]. Inflammatory cell infiltration and mediated inflammation in the kidneys are mechanisms of AKI. Neutrophils, macrophages, and CD4+ T cells have been implicated in AKI pathogenesis [28]. These cells originate from circulation and migrate into the kidney when an insult occurs [29, 30]. The SII is an inflammatory index that represents the basic innate immune status of the body. The SII obtained upon admission indicated inflammation and immune status after intracerebral hemorrhage stress. This may predict the development of AKI based on the systemic inflammatory response.

In total, 63.93% of patients reported a history of hypertension, but some patients were from rural areas and did not know their past medical histories. Therefore, the actual incidence rate of hypertension may be significantly higher than that reported. The high proportion of hypertension indicated underlying vascular injury in these patients, which may explain their increased susceptibility to AKI. Contrast media use can also result in AKI. Eight patients underwent angiography because of suspected secondary cerebral hemorrhage. Three patients presented with AKI; however, the difference between the groups was not significant. The mortality rate of patients with AKI was higher than that of patients without AKI. More importantly, the morbidity rate of patients with AKI was as high as 73.6%, indicating that more attention should be paid and additional methods should be conducted to improve the outcomes of this group as well. Renal toxic drugs, such as specific antibiotics and analgesics, should be selected elaborately, dehydrated agents should be discontinued as early as possible, and hemodynamic and fluid balance should be managed as well. Therefore, exploring novel markers for predicting AKI and outcomes in patients with AKI is valuable.

The SII can predict the development of AKI, but fails to predict the severity of AKI. Various biomarkers of AKI have been explored using different samples and methods [31], such as neutrophil gelatinase-associated lipocalin, kidney injury molecule-1, and liver fatty acid-binding protein. However, most of these biomarkers require additional testing methods and are not available for clinical application. We found that the SII and SUA were predictive markers of AKI. The SII showed a better manifestation than the SUA owing its larger AUC and superior sensitivity and specificity. SUA is also a risk factor for AKI in other clinical settings [32–34], and it is an independent risk factor for the development of stage 3 AKI in patients with spontaneous intracerebral hemorrhage [35]. SUA levels could not predict AKI severity in our study. This may be due to the more specific cohort of patients with SCH who underwent craniotomy. However, SUA levels can also be influenced by diet. The SII can be easily obtained from routine complete blood count (CBC), making it a more economical and convenient marker for the prediction of AKI. We also tested whether the SII could be a predictor of the severity of AKI, but the attempt failed. The SII was obtained from the initial CBC on admission, which indicated the baseline status of the body. The severity of AKI may be influenced by other factors, such as medications that were independent of the baseline peripheral blood cells.

The SII and SIRI could predict the outcomes of patients with AKI with SCH who underwent craniotomy. Considering the high morbidity of patients with AKI, we further investigated the possible prognostic factors in these patients. SII and SIRI values and GCS scores at admission were independent prognostic factors. The AUC of the SIRI was slightly superior to that of the SII. Similar to the SII, the SIRI was also deduced from the CBC [36]. The latter has the ability to predict the prognosis of cancers [37, 38] and spontaneous intracerebral hemorrhage [39]. Again, this indicates that the peripheral blood index can be an available candidate for prognosis. In the logistic regression models, the length of ICU stay and neutrophil, lymphocyte, and leukocyte counts were excluded. The length of ICU stay was an indicator of disease severity but not a risk factor for AKI. In addition, the calculation of the length of ICU stay was associated with the evaluation of outcomes in poor cases.

This study aimed to explore a marker on admission for predicting AKI and its prognosis. The SII and SIRI were calculated using neutrophil or lymphocyte counts, and both were leukocyte components. Multicollinearity occurred when the neutrophil, lymphocyte, and leukocyte counts and SII and SIRI values were inputted to the logistic analysis concurrently. In the multivariate regression models, the SII values were divided by 1000 to obtain an optimal OR value. The GCS is a common scale used to evaluate the consciousness level of patients with brain damage [40]. Higher GCS scores are correlated with a lower risk of poor outcomes. Therefore, the GCS curve is inversely distributed with the other indicators.

For the first time, we assessed the SII values in a specific group of patients with SCH who underwent craniotomy, which may predict the development of AKI. However, this study has some limitations: (1) this was a single-center retrospective study, which needs further verification in the future; (2) the outcomes were evaluated at the date of discharge but not a long-term period; (3) SII and SIRI values were calculated on admission without other time points; (4) the baseline SCr level was a compromise selection because real baseline SCr level was not available (the AKI was defined according to the increase in SCr by ≥0.3 mg/dl within 48 h but not increase in SCr level to ≥1.5 times baseline within the prior 7 days, and the urine output criteria were not used in the assessment of AKI); and (5) CKD3a-3b was excluded.

## Conclusions

AKI is a common complication of SCH. The SII may predict AKI in patients with SCH who underwent craniotomy; furthermore, it may predict the short-term prognosis of these patients.

## Data Availability

The data and code that support the findings of this study are available from the corresponding author upon reasonable request.

## Abbreviations

AKI: Acute kidney injury
AUC: Area under the curve
CI: Confidence interval
GCS: Glasgow Coma Scale
ICU: Intensive care unit
OR: Odds ratio
ROC: Receiver operating characteristic
SCH: Spontaneous cerebral hemorrhage
SCr: Serum creatinine
SII: Systemic immune-inflammation index
SUA: Serum uric acid
